# Supplementary material for: Magmatic connectivity among six Galápagos volcanoes revealed by satellite geodesy
Source: Nat Commun. 2023 Oct 19;14:6614. doi: 10.1038/s41467-023-42157-x (PMC10587059; doi:10.1038/s41467-023-42157-x)
Supplement: Supplementary file 1 — Supplementary Information [file 41467_2023_42157_MOESM1_ESM.pdf]

Supporting Information for “Magmatic Connectivity among Six Galápagos Volcanoes Revealed by Satellite Geodesy, Reddin et al.”

Corresponding Authors: *eeer@leeds.ac.uk*, *S.K.Ebmeier@leeds.ac.uk*

Contents:

1. Supplementary Figures 1 to 20
2. Supplementary Tables 1 and 2

Supplementary Information

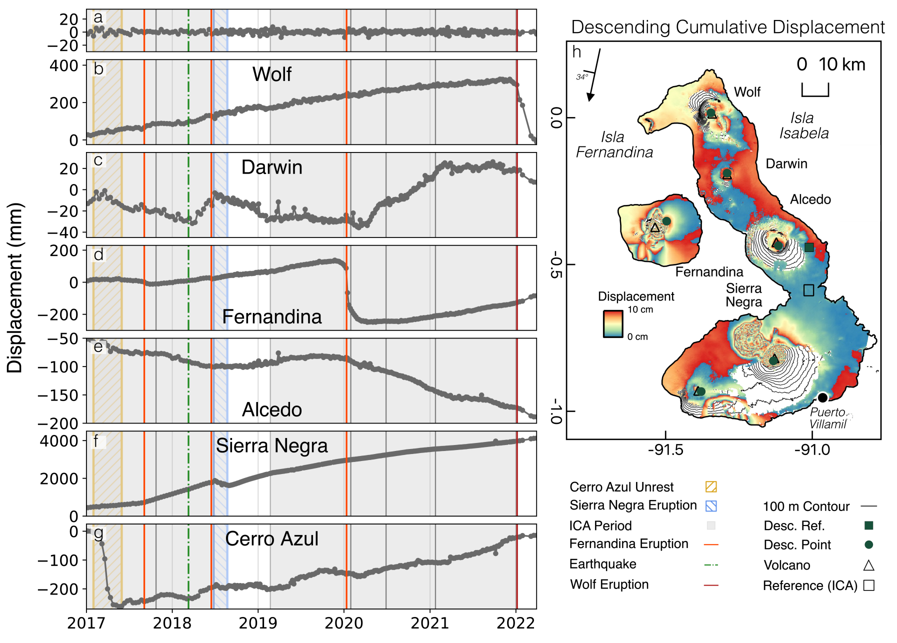

Fig. S1 Descending Sentinel-1 data for the Western Galápagos. **a**, Time series of displacement of the reference pixel, showing its variation with time. **b**–**g**, Time series of displacement at each of the major volcanoes of the Western Galápagos, from 07/01/2017–30/03/2022. Annotated are known periods of significant unrest, including unrest at Cerro Azul, and eruptions at each of Fernandina, Sierra Negra, and Wolf. The grey areas denote the periods used for Independent Component Analysis, as presented in this study. **h**, Wrapped cumulative displacement map of the Western Galápagos, across the entire time series. Each fringe corresponds to 10 cm of range change in the satellite line-of-sight. The arrow shows the satellite heading, as well as the average incidence angle. The annotated points refer to pixels used during correlation analysis, while the reference area is used during Independent Component Analysis (Figure 2).

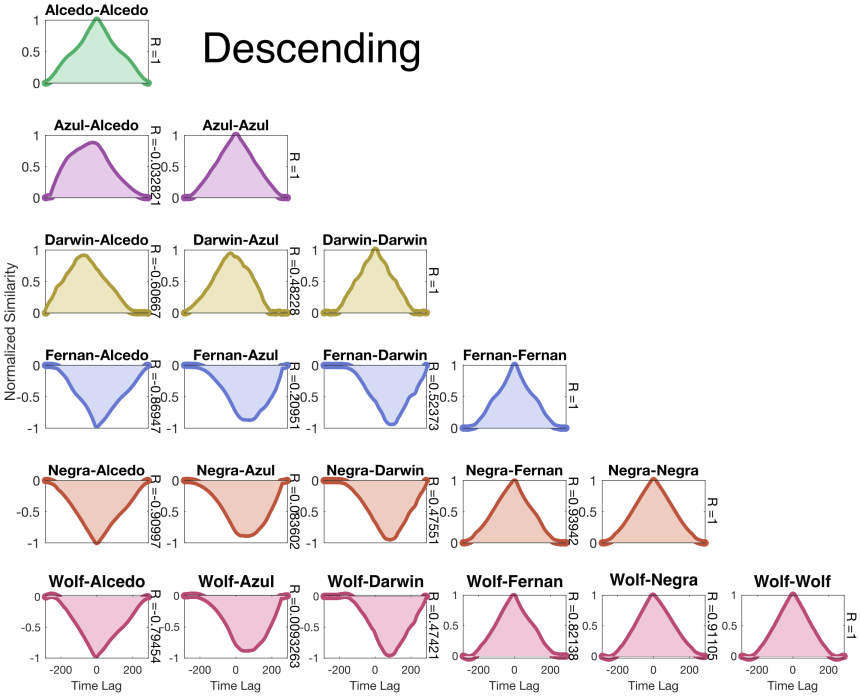

Fig. S2 Results of cross-correlation analysis for the entire time series of each volcanic pair, in Descending. Similarity between time series is presented on the y-axis, while time lag is presented on the X. The time lag refers to satellite acquisitions between time series points being compared (e.g. a lag of five means there was five satellite acquisitions between points). The correlation coefficient for each pair of time series is annotated on the right y-axis.

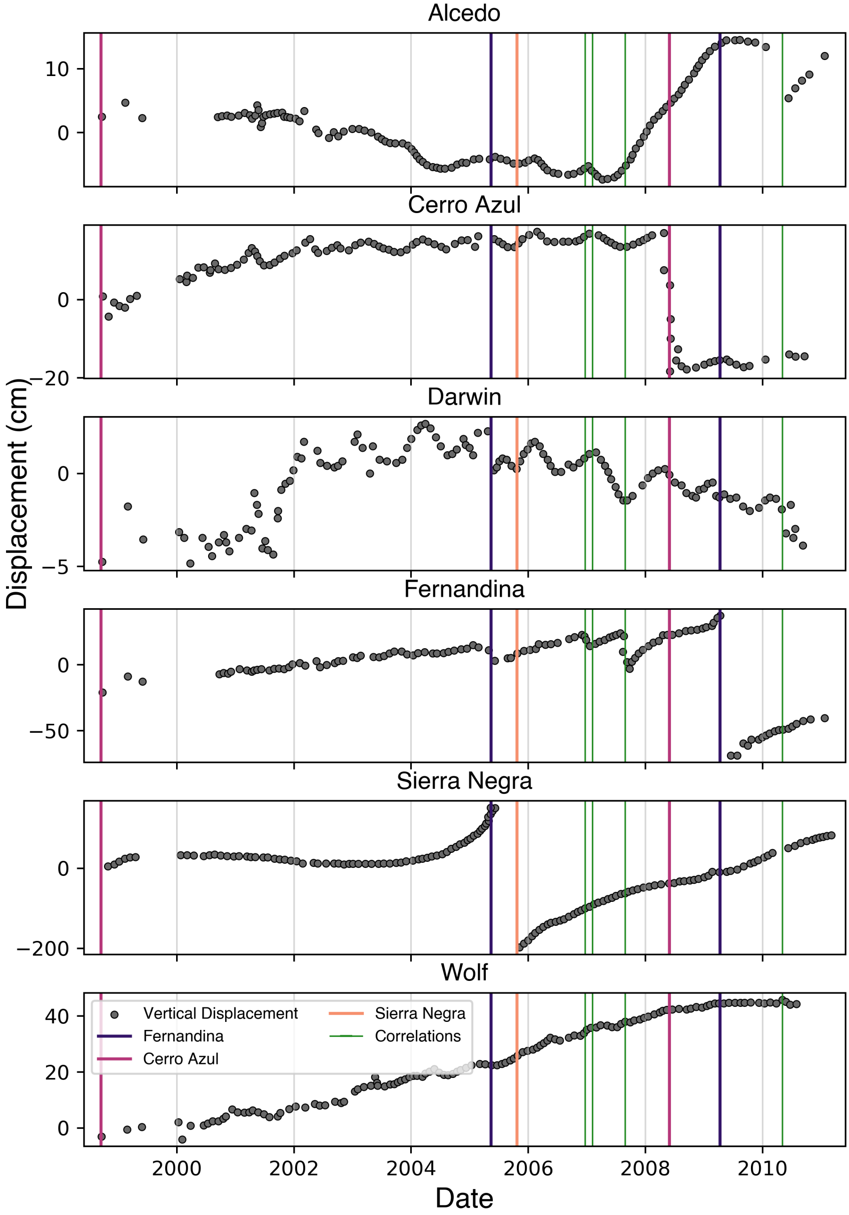

Fig. S3 Time series of displacement at each volcano in the Western Galápagos from 1998–2011, modified from [S17]. In each case, dots represent vertical displacement, in centimetres, while the vertical lines represent various eruptions. The erupted volcano is denoted by colour, while the episodes of correlated displacements observed by [S17] are marked by vertical green lines. These data are a compilation from multiple satellite missions (ERS-1/2, Envisat, Radarsat, AlOS-1.)

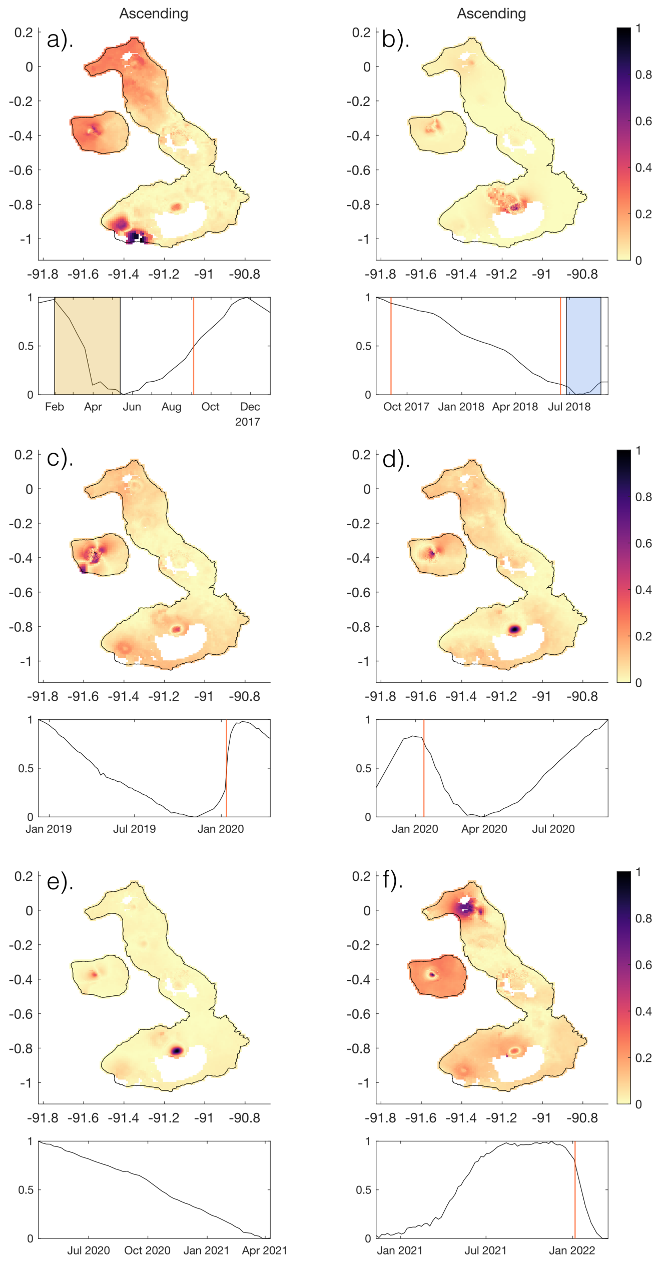

Fig. S4 Spatially reconstructed unmixing values for each independent component, in Ascending Data; the periods over which we perform ICA is informed by Figure 3. In each map, colors represent the relative strength of the retrieved independent component, providing a spatial representation of temporal data, while the lower panel shows the normalised independent component. There is some temporal overlap between each independent component, to account for overlapping windows in Figure 3. **a**, 10/15/2016–01/02/2018: 2017 unrest at Cerro Azul, and eruption of Fernandina. **b**, 08/11/2017–09/11/2018: eruptions at Fernandina in 2017 and 2018, and Sierra Negra in 2018. **c**, 12/10/2018–04/15/2020: 2020 eruption at Fernandina. **d**, 11/17/2019 09/12/2020: 2020 eruption of Fernandina. **e**, 04/15/2020–04/10/2021: no major volcanic unrest. **f**, 11/11/2020–03/18/2022: 2022 eruption of Wolf.

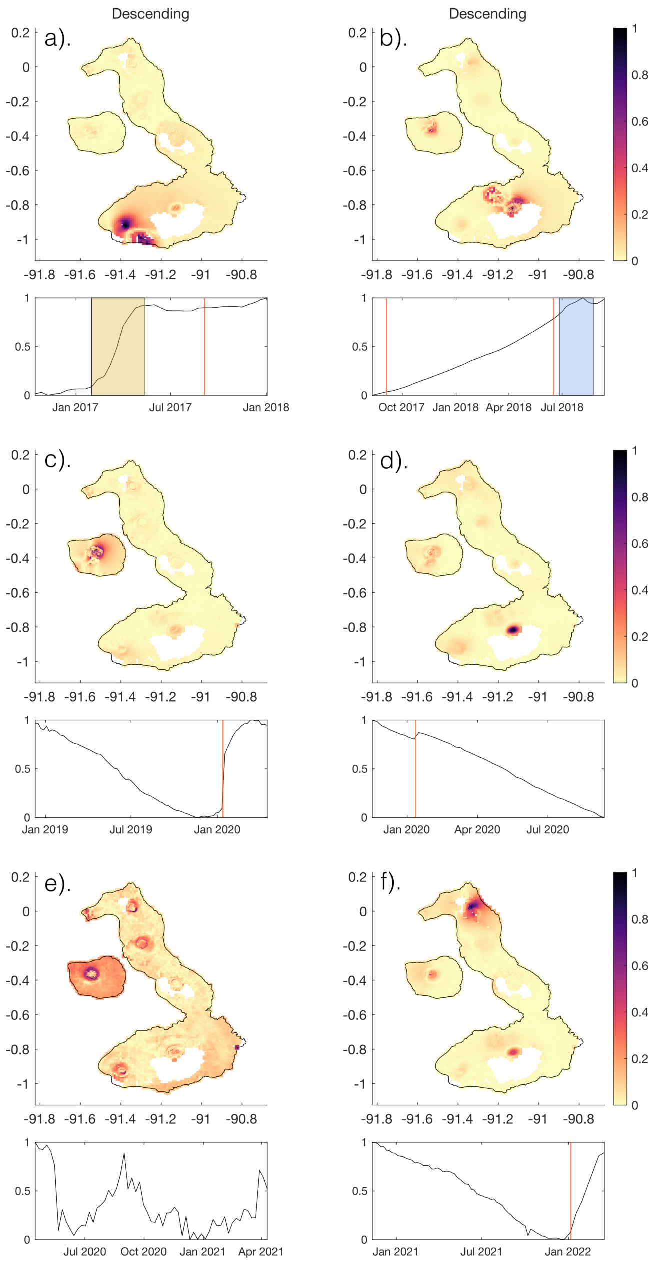

Fig. S5 Spatially reconstructed unmixing values for each independent component, in Descending Data; the periods over which we perform ICA is informed by Figure 3. In each map, colors represent the relative strength of the retrieved independent component, providing a spatial representation of temporal data, while the lower panel shows the normalised independent component. There is some temporal overlap between each independent component, to account for overlapping windows in Figure 3. **a**, 10/15/2016–01/02/2018: 2017 unrest at Cerro Azul, and eruption of Fernandina. **b**, 08/11/2017–09/11/2018: eruptions at Fernandina in 2017 and 2018, and Sierra Negra in 2018. **c**, 12/10/2018–04/15/2020: 2020 eruption at Fernandina. **d**, 11/17/2019–09/12/2020: 2020 eruption of Fernandina. **e**, 04/15/2020–04/10/2021: no major volcanic unrest. **f**, 11/11/2020–03/18/2022: 2022 eruption of Wolf.

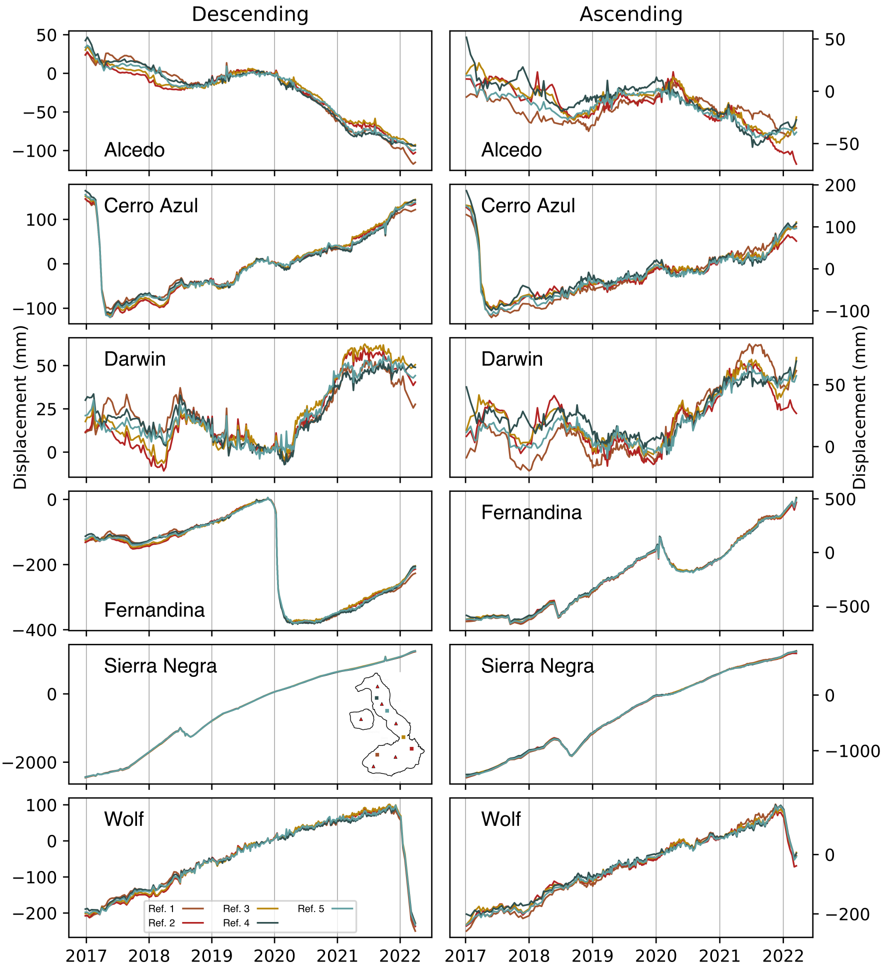

Fig. S6 Influence of choice of reference pixel, at each volcano. In each case, the plotted pixel is held constant. Descending and Ascending time series are presented in each column, while a different volcano is plotted in each row. Line colours correspond to the plotted reference pixel, the location of which is presented in the inset map.

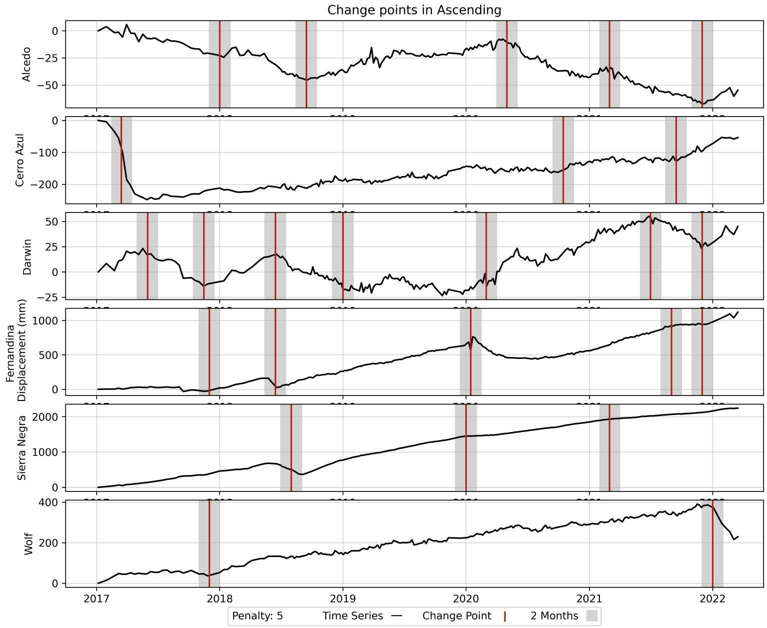

Fig. S7 Change points at each Western Galápagos volcano in the ascending track direction, from 2017–2022, as identified on the basis of change in rate or direction. Change points are annotated in red, while the grey boxes illustrate a one month period either side of the change point.

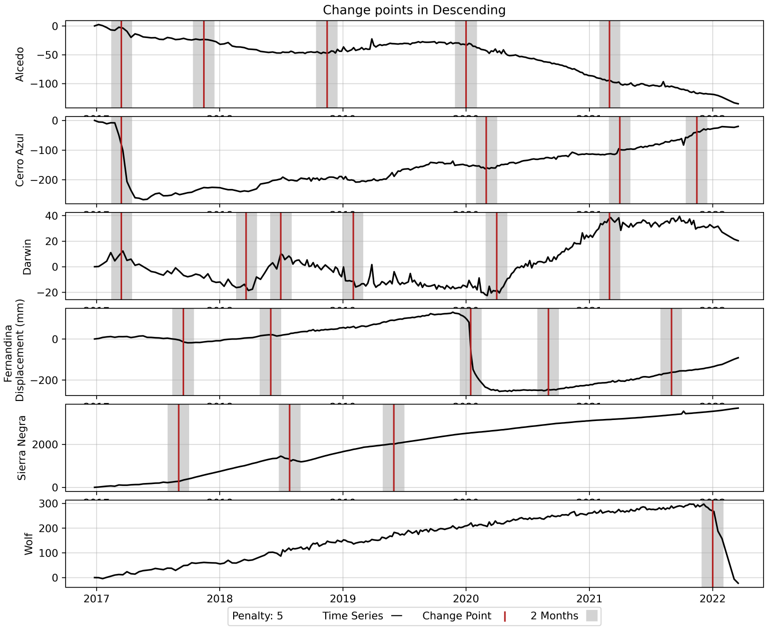

Fig. S8 Change points at each Western Galápagos volcano in the descending track direction, from 2017–2022, as identified on the basis of change in rate or direction. Change points are annotated in red, while the grey boxes illustrate a one month period either side of the change point.

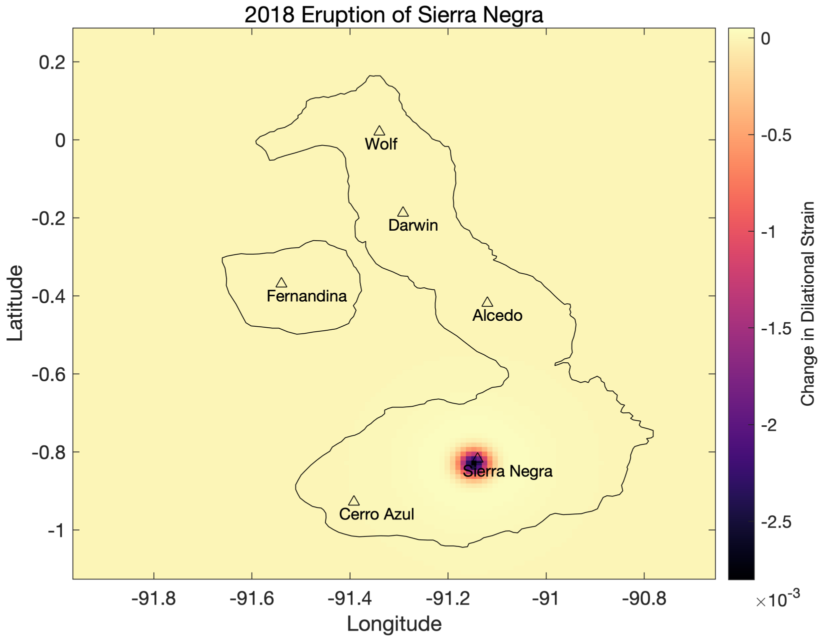

Fig. S9 Dilational strain associated with the 2018 eruption of Sierra Negra, modelled using Coulomb 3.1 [S48]. The eruption is approximated as a point source with a volume decrease of $-2x10^8\ m^3$ [S30]

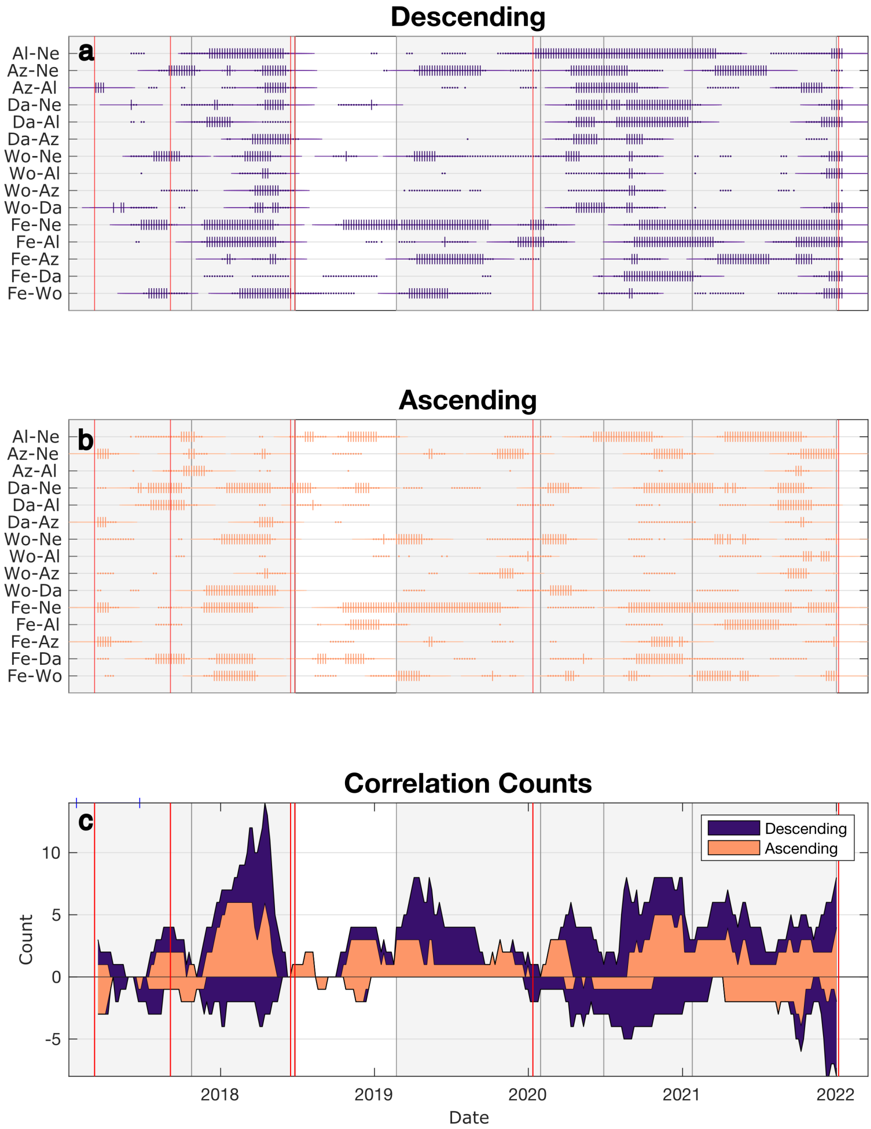

Fig. S10 Periods of heightened correlation between Western Galápagos volcanoes, determined using windowed correlation analysis, using a window size of 150 days. Red lines illustrate a significant unrest event (e.g. eruption). **a–b**, Pairwise correlation coefficients for Western Galápagos volcanoes. The location of the pixel used to create each time series is illustrated in Figure 1. Vertical lines and dots at each volcanic pair denote a respective correlation coefficients of >| 0.9 | and >| 0.75 | across the corresponding window, with each point plotted at the centre of the window. Horizontal lines illustrate the temporal extent of each window. **c**, Stacked area plot of correlation coefficients of >| 0.9 | to illustrate periods of heightened correlation. These identified periods are separated by grey boxes.

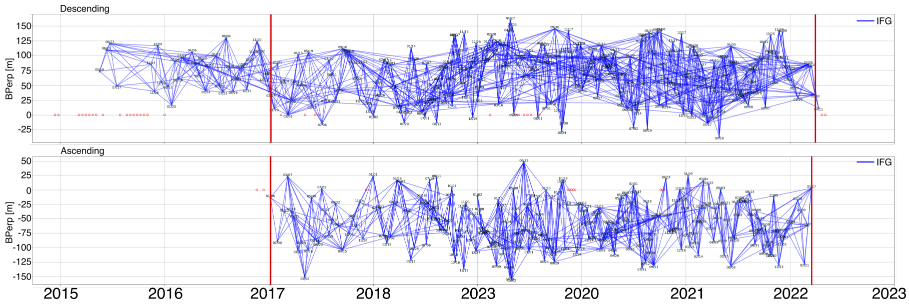

Fig. S11 Perpendicular baselines for either track direction.

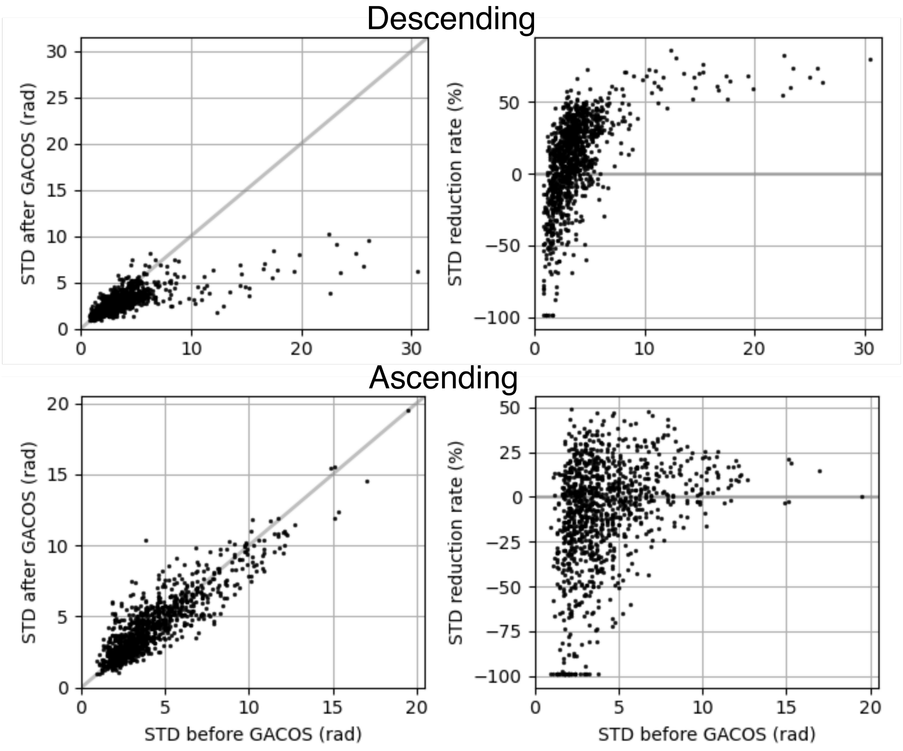

Fig. S12 Effect of GACOS correction on each data set. The left columns plot the mean standard deviation before correction against that after correction, while the right columns plot the percentage change in standard deviation due to the correction.

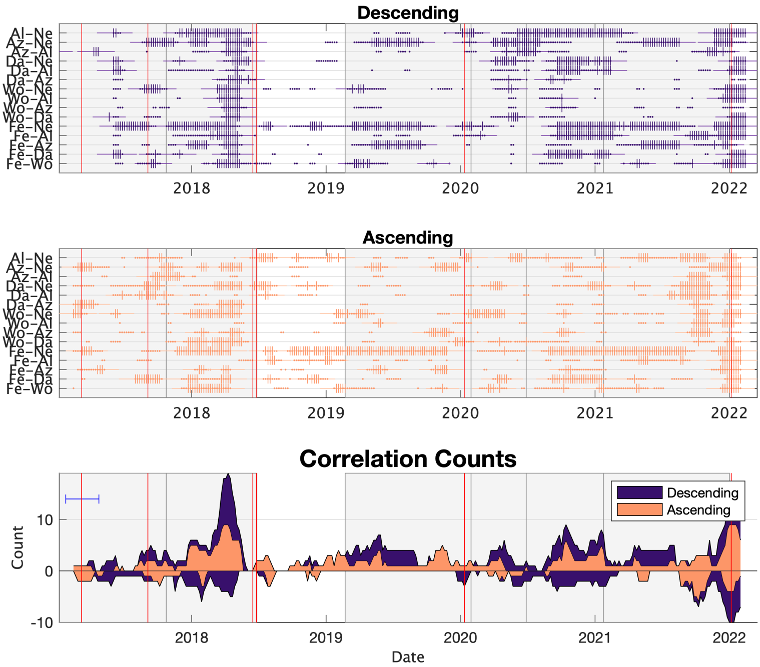

Fig. S13 Periods of heightened correlation between Western Galápagos volcanoes, determined using windowed correlation analysis. Here, with a window size of 15 acquisitions (approximately 3 months, the extent of which is shown by the horizontal black line), to show the affect of alternating the window size.

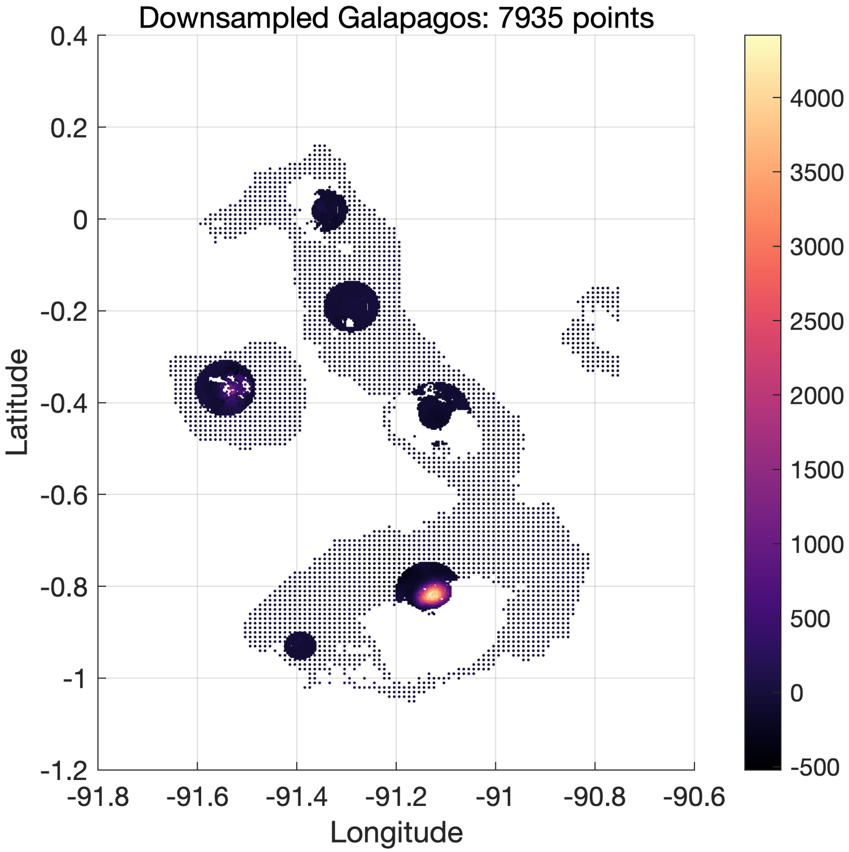

Fig. S14 The downsampled descending dataset used for Independent Component Analysis. Each point corresponds to the location of one time series of displacement

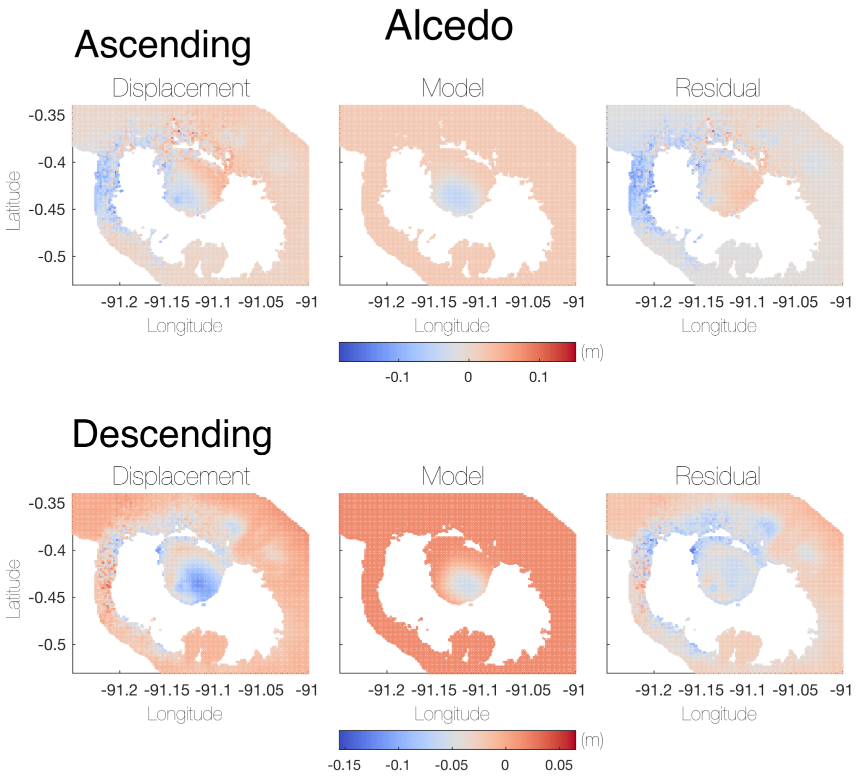

PARAM.	OPTIMAL	2.5%	97.50%
Length (m)	3328.7	1466.51	3770.22
Width (m)	4794.14	4010.34	5185.77
Depth (m)	1934.25	1665.99	3137.51
Strike (m)	207.155	198.998	220.828
X (m)	-13220.4	-13455.3	-12808.8
Y (m)	-4321.31	-4648.59	-3864.57
Opening (m)	-0.125198	-0.423341	-0.105476
InSAR Const.	0.0182488	0.0137716	0.0231403
InSAR Const.	-0.0315067	-0.0342392	-0.0274933

Fig. S15 Data, model, and residual for the best fitting sill source at Alcedo, from 26/12/2016–01/10/2021. The optimal source parameters, as well as the 2.5% and 97.5% bounds are presented in the underlying table.

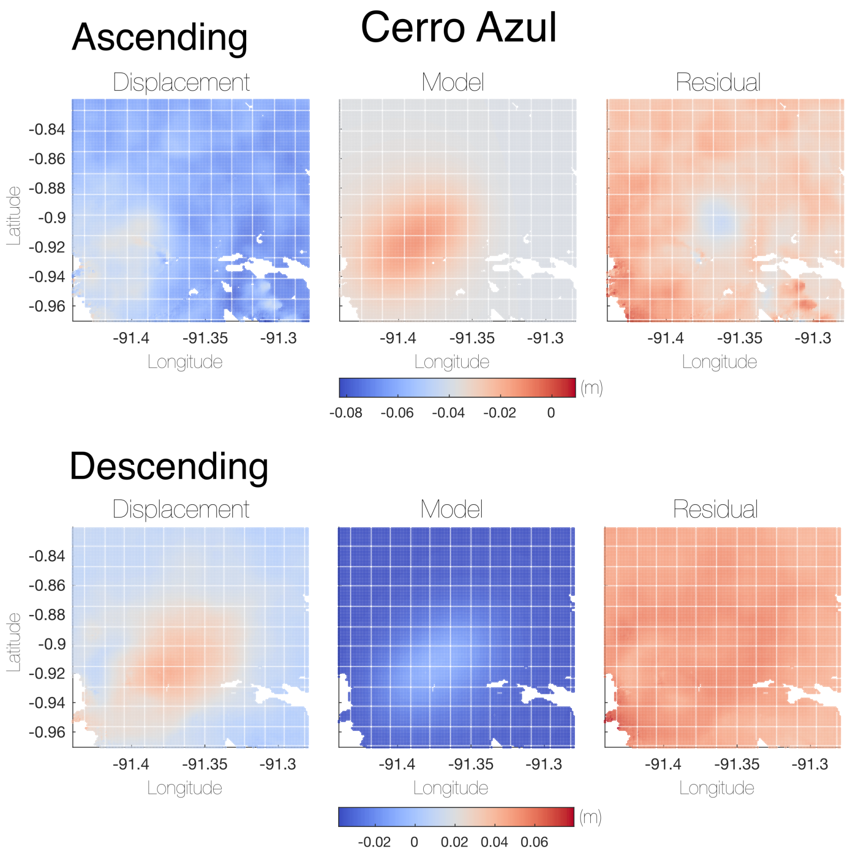

PARAM.	OPTIMAL	2.5%	97.50%
Length (m)	271.185	150.162	313.129
Width (m)	7678.99	7541.83	7771.15
Depth (m)	4522.96	4303.95	4518.23
Strike (m)	145.552	145.081	146.86
X (m)	2944.73	2858.59	2987.62
Y (m)	-4627.32	-4672.01	-4557.39
Opening (m)	1.04704	0.880844	1.71992
InSAR Const.	-0.0382424	-0.0379602	-0.0316358
InSAR Const.	0.0115861	0.0116052	0.012296

Fig. S16 Data, model, and residual for the best fitting sill source at Cerro Azul, from 02/02/2020–03/01/2021. The optimal source parameters, as well as the 2.5% and 97.5% bounds are presented in the underlying table.

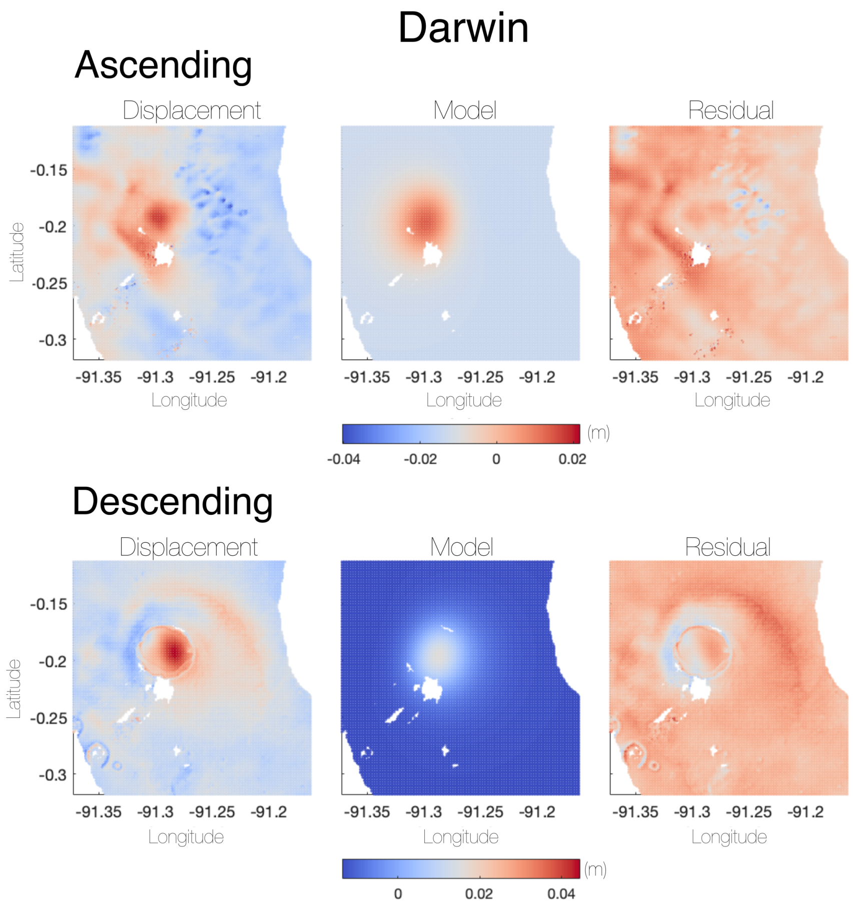

PARAM.	OPTIMAL	2.5%	97.50%
Length (m)	188.77	124.452	1051.43
Width (m)	3643.94	1259.22	4492.73
Depth (m)	4264.53	4015.04	4733.31
Strike (m)	105.663	90.3973	284.478
X (m)	2627.74	2282.73	3442.42
Y (m)	9519.86	9003.75	12161.7
Opening (m)	2.13353	0.46785	4.00451
InSAR Const.	-0.0134238	-0.014303	-0.0127242
InSAR Const.	0.0096809	0.00864868	0.01064

Fig. S17 Data, model, and residual for the best fitting sill source at Darwin, from 01/07/2020–28/03/2021. The optimal source parameters, as well as the 2.5% and 97.5% bounds are presented in the underlying table.

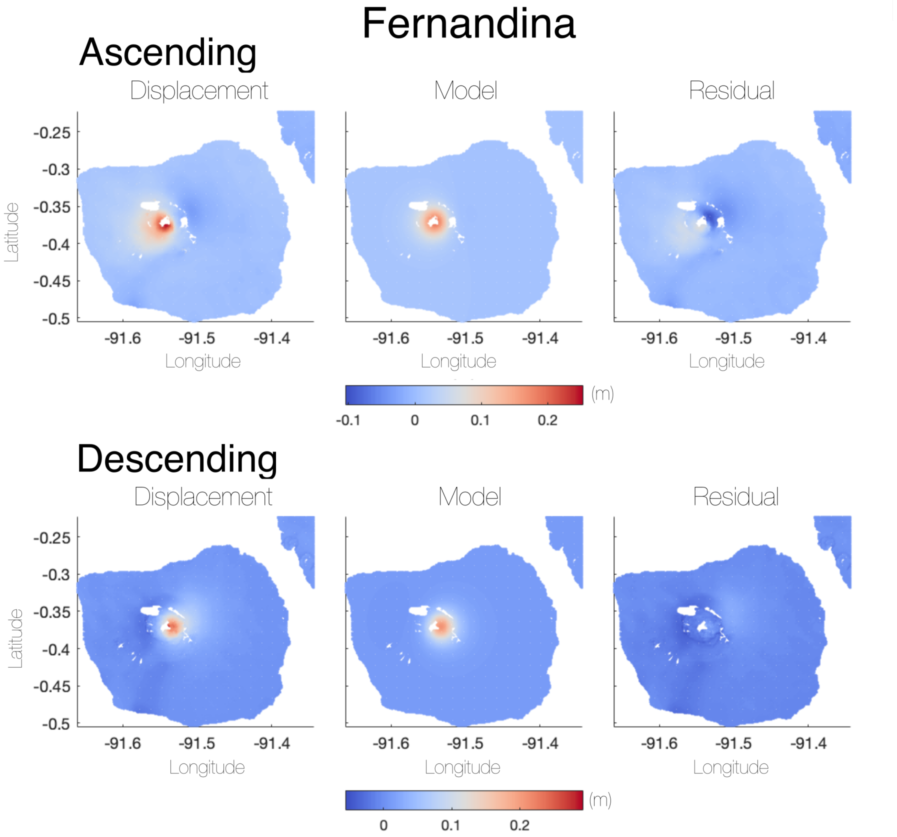

PARAM.	OPTIMAL	2.5%	97.50%
Length (m)	896.577	707.449	1260.96
Width (m)	108.212	111.66	271.713
Depth (m)	3106.91	3076.91	3140.76
Strike (m)	51.4147	44.9203	57.6794
X (m)	12757.5	12754.9	12822.6
Y (m)	-339.407	-404.027	-335.622
Opening (m)	49.16	14.139	49.6947
InSAR Const.	0.00971126	0.00922391	0.0104413
InSAR Const.	0.00482871	0.00456167	0.00502437

Fig. S18 Data, model, and residual for the best fitting sill source at Fernandina, from 01/07/2020–28/03/2021. The optimal source parameters, as well as the 2.5% and 97.5% bounds are presented in the underlying table.

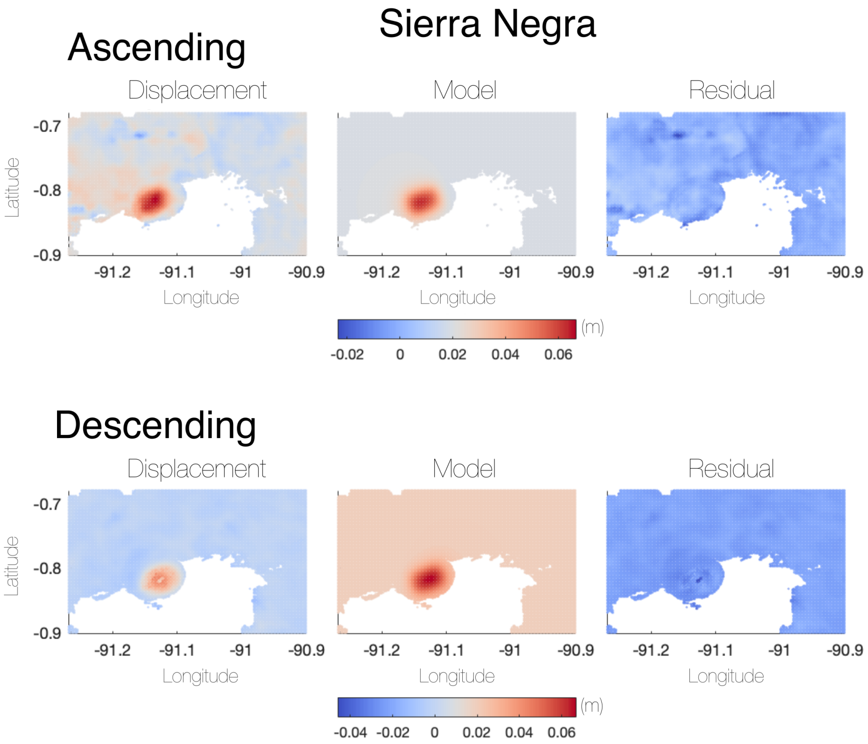

PARAM.	OPTIMAL	2.5%	97.50%
Length (m)	128.048	104.925	923.512
Width (m)	4553.33	4123.59	4930.47
Depth (m)	1957.84	1796.54	2082.25
Strike (m)	218.158	214.464	221.842
X (m)	13783	13417.1	14179.7
Y (m)	-6381.72	-6582.94	-6222.33
Opening (m)	1.05408	0.14301	1.3071
InSAR Const.	-0.0086843	-0.0093517	-0.0077237
InSAR Const.	0.00391561	0.00308953	0.00466889

Fig. S19 Data, model, and residual for the best fitting sill source at Sierra Negra, from 29/12/2020–03/02/2021. The optimal source parameters, as well as the 2.5% and 97.5% bounds are presented in the underlying table.

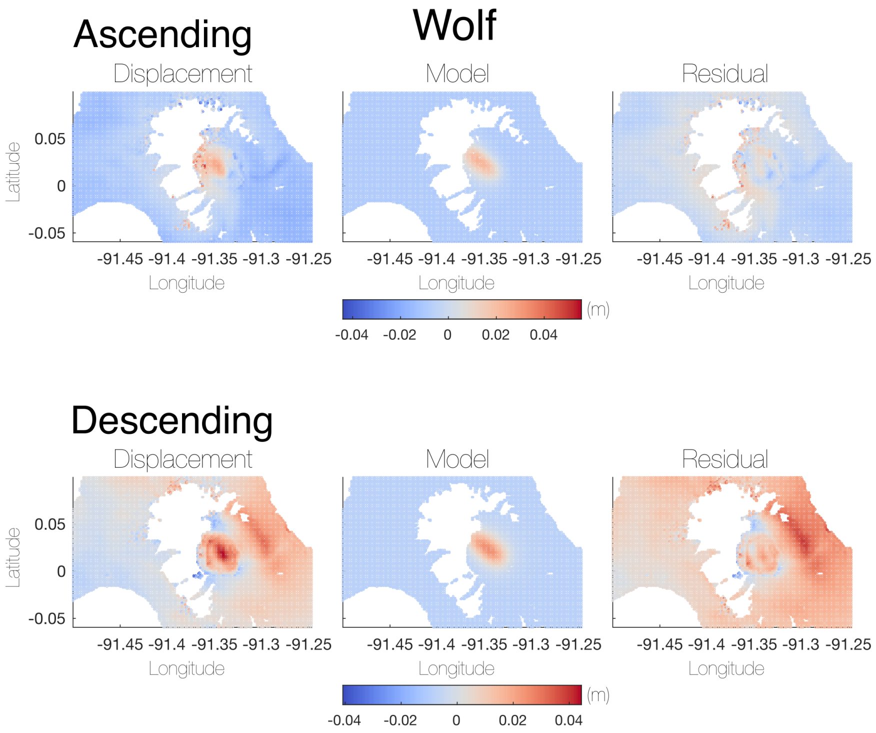

PARAM.	OPTIMAL	2.5%	97.50%
Length (m)	128.048	104.925	923.512
Width (m)	4553.33	4123.59	4930.47
Depth (m)	1957.84	1796.54	2082.25
Strike (m)	218.158	214.464	221.842
X (m)	13783	13417.1	14179.7
Y (m)	-6381.72	-6582.94	-6222.33
Opening (m)	1.05408	0.14301	1.3071
InSAR Const.	-0.0086843	-0.0093517	-0.0077237
InSAR Const.	0.00391561	0.00308953	0.00466889

Fig. S20 Data, model, and residual for the best fitting sill source at Wolf, from 01/06/2020–27/05/2021. The optimal source parameters, as well as the 2.5% and 97.5% bounds are presented in the underlying table.

07/01/2017	27/03/2018	28/12/2018	31/08/2019	21/04/2020	29/11/2020	09/07/2021
19/01/2017	08/04/2018	03/01/2019	06/09/2019	27/04/2020	05/12/2020	15/07/2021
31/01/2017	20/04/2018	09/01/2019	12/09/2019	03/05/2020	11/12/2020	21/07/2021
12/02/2017	02/05/2018	15/01/2019	18/09/2019	09/05/2020	17/12/2020	27/07/2021
24/02/2017	14/05/2018	21/01/2019	30/09/2019	15/05/2020	23/12/2020	02/08/2021
08/03/2017	26/05/2018	27/01/2019	06/10/2019	21/05/2020	29/12/2020	08/08/2021
20/03/2017	07/06/2018	02/02/2019	12/10/2019	27/05/2020	04/01/2021	14/08/2021
01/04/2017	19/06/2018	08/02/2019	18/10/2019	02/06/2020	10/01/2021	20/08/2021
13/04/2017	01/07/2018	20/02/2019	24/10/2019	08/06/2020	16/01/2021	26/08/2021
25/04/2017	07/07/2018	26/02/2019	30/10/2019	14/06/2020	22/01/2021	01/09/2021
07/05/2017	13/07/2018	04/03/2019	05/11/2019	20/06/2020	28/01/2021	07/09/2021
19/05/2017	25/07/2018	10/03/2019	17/11/2019	26/06/2020	03/02/2021	13/09/2021
31/05/2017	31/07/2018	16/03/2019	23/11/2019	02/07/2020	09/02/2021	19/09/2021
12/06/2017	06/08/2018	22/03/2019	29/11/2019	08/07/2020	15/02/2021	25/09/2021
24/06/2017	12/08/2018	28/03/2019	05/12/2019	14/07/2020	21/02/2021	01/10/2021
06/07/2017	18/08/2018	03/04/2019	11/12/2019	20/07/2020	27/02/2021	07/10/2021
18/07/2017	24/08/2018	09/04/2019	17/12/2019	26/07/2020	05/03/2021	13/10/2021
30/07/2017	30/08/2018	15/04/2019	23/12/2019	01/08/2020	11/03/2021	19/10/2021
11/08/2017	05/09/2018	21/04/2019	29/12/2019	07/08/2020	17/03/2021	25/10/2021
23/08/2017	11/09/2018	27/04/2019	04/01/2020	13/08/2020	23/03/2021	31/10/2021
04/09/2017	17/09/2018	03/05/2019	10/01/2020	19/08/2020	29/03/2021	06/11/2021
16/09/2017	23/09/2018	09/05/2019	16/01/2020	25/08/2020	04/04/2021	12/11/2021
28/09/2017	29/09/2018	21/05/2019	22/01/2020	31/08/2020	10/04/2021	18/11/2021
10/10/2017	05/10/2018	27/05/2019	28/01/2020	06/09/2020	16/04/2021	24/11/2021
22/10/2017	11/10/2018	02/06/2019	03/02/2020	12/09/2020	22/04/2021	06/12/2021
03/11/2017	17/10/2018	14/06/2019	09/02/2020	18/09/2020	28/04/2021	12/12/2021
15/11/2017	23/10/2018	20/06/2019	15/02/2020	24/09/2020	04/05/2021	18/12/2021
27/11/2017	29/10/2018	26/06/2019	21/02/2020	30/09/2020	10/05/2021	24/12/2021
09/12/2017	04/11/2018	02/07/2019	27/02/2020	06/10/2020	16/05/2021	05/01/2022
21/12/2017	10/11/2018	14/07/2019	04/03/2020	12/10/2020	22/05/2021	17/01/2022
02/01/2018	16/11/2018	20/07/2019	10/03/2020	18/10/2020	28/05/2021	29/01/2022
14/01/2018	22/11/2018	26/07/2019	16/03/2020	24/10/2020	03/06/2021	06/03/2022
26/01/2018	28/11/2018	01/08/2019	22/03/2020	30/10/2020	09/06/2021	18/03/2022
07/02/2018	04/12/2018	07/08/2019	28/03/2020	05/11/2020	15/06/2021	30/03/2022
19/02/2018	10/12/2018	13/08/2019	03/04/2020	11/11/2020	21/06/2021	
03/03/2018	16/12/2018	19/08/2019	09/04/2020	17/11/2020	27/06/2021	
15/03/2018	22/12/2018	25/08/2019	15/04/2020	23/11/2020	03/07/2021	

Table S1 Dates used in the Descending data set (256 in total). These data are taken from both Sentinel-1A and Sentinel-1B, using orbit number 128, covering the islands of both Fernandina and Isabela

06/01/2017	01/05/2018	14/01/2019	30/08/2019	26/04/2020	22/12/2020	13/08/2021
30/01/2017	13/05/2018	20/01/2019	05/09/2019	02/05/2020	28/12/2020	19/08/2021
23/02/2017	25/05/2018	26/01/2019	11/09/2019	08/05/2020	03/01/2021	25/08/2021
07/03/2017	18/06/2018	01/02/2019	17/09/2019	14/05/2020	09/01/2021	31/08/2021
19/03/2017	30/06/2018	19/02/2019	23/09/2019	20/05/2020	15/01/2021	06/09/2021
31/03/2017	06/07/2018	25/02/2019	29/09/2019	26/05/2020	27/01/2021	12/09/2021
12/04/2017	12/07/2018	03/03/2019	05/10/2019	01/06/2020	02/02/2021	18/09/2021
24/04/2017	18/07/2018	09/03/2019	11/10/2019	07/06/2020	08/02/2021	24/09/2021
06/05/2017	24/07/2018	15/03/2019	23/10/2019	13/06/2020	14/02/2021	30/09/2021
18/05/2017	30/07/2018	21/03/2019	29/10/2019	19/06/2020	20/02/2021	06/10/2021
30/05/2017	05/08/2018	27/03/2019	04/11/2019	25/06/2020	26/02/2021	12/10/2021
11/06/2017	11/08/2018	02/04/2019	10/11/2019	01/07/2020	04/03/2021	18/10/2021
23/06/2017	17/08/2018	08/04/2019	16/12/2019	07/07/2020	10/03/2021	24/10/2021
05/07/2017	23/08/2018	14/04/2019	22/12/2019	13/07/2020	16/03/2021	30/10/2021
17/07/2017	04/09/2018	20/04/2019	28/12/2019	25/07/2020	22/03/2021	05/11/2021
29/07/2017	16/09/2018	26/04/2019	03/01/2020	31/07/2020	28/03/2021	11/11/2021
10/08/2017	22/09/2018	02/05/2019	09/01/2020	12/08/2020	09/04/2021	17/11/2021
22/08/2017	28/09/2018	08/05/2019	15/01/2020	18/08/2020	15/04/2021	23/11/2021
03/09/2017	04/10/2018	14/05/2019	21/01/2020	24/08/2020	21/04/2021	29/11/2021
15/09/2017	10/10/2018	20/05/2019	27/01/2020	30/08/2020	27/04/2021	05/12/2021
09/10/2017	16/10/2018	26/05/2019	02/02/2020	05/09/2020	03/05/2021	11/12/2021
21/10/2017	22/10/2018	01/06/2019	08/02/2020	11/09/2020	09/05/2021	17/12/2021
02/11/2017	28/10/2018	13/06/2019	14/02/2020	17/09/2020	15/05/2021	04/01/2022
14/11/2017	03/11/2018	19/06/2019	20/02/2020	29/09/2020	21/05/2021	28/01/2022
26/11/2017	09/11/2018	25/06/2019	26/02/2020	23/10/2020	27/05/2021	09/02/2022
01/01/2018	15/11/2018	01/07/2019	03/03/2020	29/10/2020	08/06/2021	21/02/2022
13/01/2018	21/11/2018	07/07/2019	09/03/2020	04/11/2020	14/06/2021	05/03/2022
25/01/2018	27/11/2018	13/07/2019	15/03/2020	10/11/2020	20/06/2021	17/03/2022
06/02/2018	03/12/2018	19/07/2019	21/03/2020	16/11/2020	26/06/2021	
18/02/2018	09/12/2018	25/07/2019	27/03/2020	22/11/2020	02/07/2021	
02/03/2018	21/12/2018	31/07/2019	02/04/2020	28/11/2020	08/07/2021	
14/03/2018	27/12/2018	06/08/2019	08/04/2020	04/12/2020	14/07/2021	
26/03/2018	02/01/2019	18/08/2019	14/04/2020	10/12/2020	26/07/2021	
07/04/2018	08/01/2019	24/08/2019	20/04/2020	16/12/2020	07/08/2021	

Table S2 Dates used in the Ascending data set (204 in total). These data are taken from both Sentinel-1A and Sentinel-1B, using orbit number 106, covering the islands of both Fernandina and Isabela
